# The clinical value of immune cell composition in the diagnosis of pediatric sepsis

**DOI:** 10.3389/fped.2025.1599694

**Published:** 2025-07-18

**Authors:** Jie Shen, Qing Zhong, Jing Su, Xuan Shen, Wenya Li, Tianyu Sun, Yaoyun Zhang, Jijin Qi

**Affiliations:** ^1^ Department of Clinical Laboratory, Jiangsu Province (Suqian) Hospital, Suqian, Jiangsu, China; ^2^ Department of Hematology Laboratory, Jiangsu Province (Suqian) Hospital, Suqian, Jiangsu, China

**Keywords:** sepsis, immune cells, infection, NLR, ROC

## Abstract

**Background:**

The objective of this study was to evaluate the diagnostic significance of various immune cell ratios in children with sepsis via the analysis of clinical data.

**Methods:**

This retrospective study analyzed clinical data admitted to the pediatric intensive care unit (PICU) of Jiangsu Province (Suqian) Hospital between January 2023 and December 2024. Receiver operating characteristic (ROC) curve analysis evaluated the diagnostic performance of peripheral blood laboratory assessments and Immune cell ratios (platelet count divided by lymphocyte count (PLR), monocyte count divided by lymphocyte count (MLR), neutrophil count divided by lymphocyte count (NLR), the ratio of neutrophils to (white blood cells—neutrophils) (dNLR), and neutrophil count multiplied by 100 divided by (lymphocyte count multiplied by platelet count) (NLPR)). Using Statistical Package for the Social Sciences (SPSS) 26.0 performed by Mann–Whitney U and Chi-square tests, with significance defined as *P* < 0.05.

**Result:**

We analyzed the clinical data from 321 pediatric patients [201 sepsis (SP) and 120 healthy controls (HC)]. Compared to HC, the SP group exhibited elevated white blood cell (WBC), neutrophils, procalcitonin (PCT), platelets (PLT), and erythrocyte sedimentation rate (ESR) but reduced lymphocytes, monocytes, and hemoglobin; C-reactive protein (CRP) was significantly increased in the SP group (*P* < 0.001). ROC analysis identified NLR [The area under the ROC curve (AUC) = 0.932], PLR (AUC = 0.907), and NLPR (AUC = 0.848) as effective immune ratio biomarkers, while MLR (AUC = 0.784) showed limited utility. Among laboratory tests, WBC (AUC = 0.902), neutrophils (AUC = 0.919), CRP (AUC = 0.923), PLT (AUC = 0.879), and ESR (AUC = 0.875) demonstrated strong diagnostic accuracy, whereas lymphocyte, monocyte, and hemoglobin levels were less discriminative.

**Conclusion:**

Our study reveals that combining immune cell ratios and CRP significantly enhances early detection of pediatric sepsis, improving clinical outcomes.

## Introduction

Pediatric sepsis is a complex and life-threatening disease characterized by organ dysfunction resulting from a disordered host response to infection in children. It is a leading cause of multi-organ failure, affecting around 5.3 million people worldwide each year, especially children (1, 2). The disease often presents with rapid changes and atypical symptoms, making diagnosis challenging (3). Therefore, timely recognition and intervention are essential to improve outcomes and save lives.

Immune cells play a crucial role in the initiation and progression of sepsis. They engage in both innate and adaptive immune responses through life activities such as phagocytosis, the release of reactive oxygen species, and cytokine production (4, 5). Current research indicates that sepsis can affect the number and function of immune cells (6–8). With underdeveloped immune systems and limited regulatory and compensatory abilities, children are more susceptible to immune cell imbalances than adults (9–11). These findings underscore the importance of understanding the unique vulnerabilities of pediatric immune systems in the context of sepsis to develop targeted therapeutic strategies.

Recent research highlights immune cell profiles and inflammatory markers as important for understanding the severity of sepsis and its outcomes in children. Neutrophils are found to exhibit various dysfunctions at the late stage of sepsis, including impaired apoptosis, severely damaged chemotaxis, and extensive tissue infiltration (12–16). In adaptive immunity, sepsis-induced apoptosis in septic shock patients causes lymphopenia, affecting all T cell subsets (CD4+, CD8+, and natural killer cells) except T regulatory cells, which promotes immunosuppression (7, 17–19). Decreased percentages of CD4 T cells and less diverse T-cell receptor repertoires were reported in pediatric sepsis patients (20–22). Various studies have indicated that immune cell ratios, such as the neutrophil-to-lymphocyte ratio (NLR) and the monocyte-to-lymphocyte ratio (MLR), may correlate with disease severity and patient outcomes, suggesting their potential utility in clinical practice (23). Changes in immune cells are common in sepsis patients, yet limited research exists on the association between immune cell ratios and the diagnosis of septic children.

Despite these promising findings, there remains a critical gap in the application of these biomarkers in routine clinical settings. Variations in patient responses and the complex nature of immune reactions to infections make it difficult to establish standardized diagnostic tools. There is a pressing need for studies that systematically evaluate the diagnostic value of clinical outcomes in pediatric sepsis. The objective of this study was to explore the value of various immune cell ratios for the early diagnosis of septic children, aiming to provide reliable insights for the early identification of patients.

## Method

### Study design

This retrospective study analyzed clinical data from pediatric patients admitted to the PICU of the Jiangsu Province (Suqian) Hospital between January 2023 and December 2024. A total of 321 children were included in the analysis of immune cell proportions, with 201 assigned to the sepsis group (SP) and 120 to the healthy control group (HC). The study was approved by the ethics committee of Jiangsu Province (Suqian) Hospital, and informed consent was obtained from all parents or legal guardians of the children.

### Inclusion and exclusion criteria

Inclusion criteria: (1) Patients identified with sepsis upon entry to the hospital; (2) Aged between 28 days and 18 years; (3) Only data from the initial admission to the hospital were considered for patients with multiple admissions.

Exclusion criteria: (1) Absence of complete blood count data within 24 h of admission to the hospital; (2) Patients with hematological cancers or terminal malignant neoplasms; (3) Patients who had received organ transplantation or utilized hormones, cytotoxic agents, or immunosuppression within the preceding 2 weeks; (4) Patients with a hospital duration of less than 24 h; (5) Patients who opted to cease treatment, voluntarily discharged themselves, or were transferred to other healthcare facilities.

### Data collection

Clinical information of patients with pediatric sepsis was collected from the medical record management system and entered and managed using Epidata V.3.1 software. Baseline demographics, including age, gender, comorbidities and medication intake were recorded. Peripheral blood samples were collected for laboratory assessments, including white blood cell (WBC) count, lymphocyte count, neutrophil count, monocyte count, hemoglobin, platelet count, C-reactive protein (CRP), procalcitonin (PCT), and erythrocyte sedimentation rate (ESR).

Regarding the immune cell ratios, the following calculation methods were applied in this study: (1) PLR = platelet count divided by lymphocyte count; (2) MLR = monocyte count divided by lymphocyte count; (3) NLR = neutrophil count divided by lymphocyte count; (4) dNLR = the ratio of neutrophils to (white blood cells—neutrophils); and (5) NLPR = neutrophil count multiplied by 100 divided by (lymphocyte count multiplied by platelet count).

### Statistical analysis

Continuous variables were analyzed using the Mann–Whitney U non-parametric test, whereas categorical variables between SP and HC groups were compared through the Chi-square test. Receiver operating characteristic (ROC) curve analysis was conducted using SPSS 26.0 (IBM SPSS Statistics, USA) to evaluate the diagnostic potential of peripheral blood immune cell ratios. The area under the ROC curve (AUC) with its exact binomial 95% confidence interval (CI) was calculated, and its statistical significance against the null hypothesis value (AUC = 0.5) was determined using the *Z*-test. Diagnostic accuracy was categorized as excellent when AUC exceeded 0.9. Optimal cutoff values were derived by maximizing the Youden index (sensitivity + specificity—1), a composite metric reflecting the trade-off between true positive identification and false positive avoidance. Corresponding sensitivity, specificity, and associated 95% CIs were reported. All statistical tests were two-tailed, with significance defined as *P* < 0.05. Data processing adhered to rigorous methodological standards for clinical diagnostic studies.

## Result

### General information of the enrolled patients

**Figure 1:**
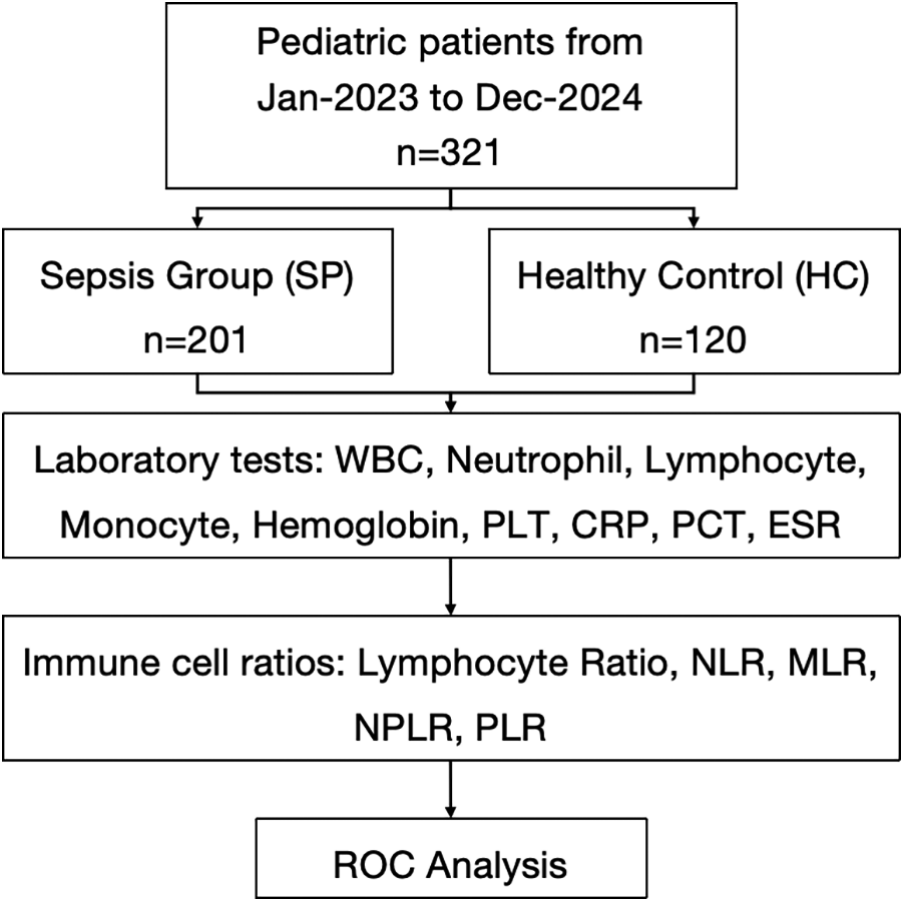
Flowchart of the study.

A total of 321 participants were included in the study to evaluate the laboratory test for sepsis detection. 201 participants (62.6%) were assigned to the SP group with an average age of 2.71 [Standard Deviation (SD) 2.48] years and 56.7% of the participants were male, and 120 participants (37.4%) were allocated to the HC group with a mean age of 2.29 (SD 1.03) years and 62.0% of the participants were male. No significant differences in age and gender were observed between the groups (*P* = 0.708, *P* = 0.891) (Figure 1).

### Comparison of the laboratory tests between SP group and HC group

As for the laboratory tests, significant differences were found between the two groups across various clinical and laboratory parameters. The SP group had significantly higher levels of WBC count (23.3 vs. 7.2, *P* < 0.001), neutrophil count (17.2 vs. 2.3, *P* < 0.001), monocyte counts (1.2 vs. 0.9, *P* < 0.001), PCT (0.55 vs. 0.05, *P* < 0.001), PLT (324 vs. 202, *P* < 0.001), CRP (61.7 vs. 4.3, *P* < 0.001) and ESR (44.1 vs. 7.0, *P* < 0.001) compared to the HC group. Conversely, the SP group showed significantly lower levels of lymphocyte count (2.3 vs. 3.7, *P* < 0.001) (Table 1).

**Table 1:** The clinical outcomes and baseline demographics between SP and HC.

Variable	SP (*n* = 201)	HC (*n* = 120)	*P*
Age, years	2.71 ± 2.48	2.29 ± 1.03	0.708
Gender, *n* (%)			0.891
Male	114 (56.7%)	31 (62.0%)	
Female	87 (43.3%)	19 (38.0%)	
Laboratory tests			
WBC × 10^9^/L	23.3 (17.9, 29.8)	7.2 (6.3, 8.9)	<0.001
Neutrophil × 10^9^/L	17.2 (12.1, 25.0)	2.3 (1.9, 3.1)	<0.001
Lymphocyte × 10^9^/L	2.3 (1.5, 3.2)	3.7 (2.9, 4.2)	<0.001
Monocyte × 10^9^/L	1.2 (0.8, 1.7)	0.9 (0.6, 1.2)	<0.001
Hemoglobin × g/L	123 (115, 129)	128 (119, 137)	<0.054
PLT × 10^9^/L	324 (264, 397)	202 (147, 251)	<0.001
CRP mg/L	61.7 (30.2, 88.8)	4.3 (2.4, 7.3)	<0.001
PCT μg/L	0.55 (0.17, 1.79)	0.05 (0.02, 0.42)	<0.001
ESR mm/h	44.1 (16.6, 67.8)	7.0 (3.4, 9.5)	<0.001
Lymphocyte ratio			
NLR	7.25 (4.05, 11.63)	0.66 (0.53, 0.80)	<0.001
MLR	0.49 (0.35, 0.75)	0.26 (0.18, 0.39)	<0.001
NLPR	0.06 (0.03, 0.08)	0.01 (0.01, 0.02)	<0.001
PLR	135.37 (102.75, 220.32)	55.86 (44.04, 74.96)	<0.001

### Efficacy evaluation of immune cell proportions

**Figure 2:**
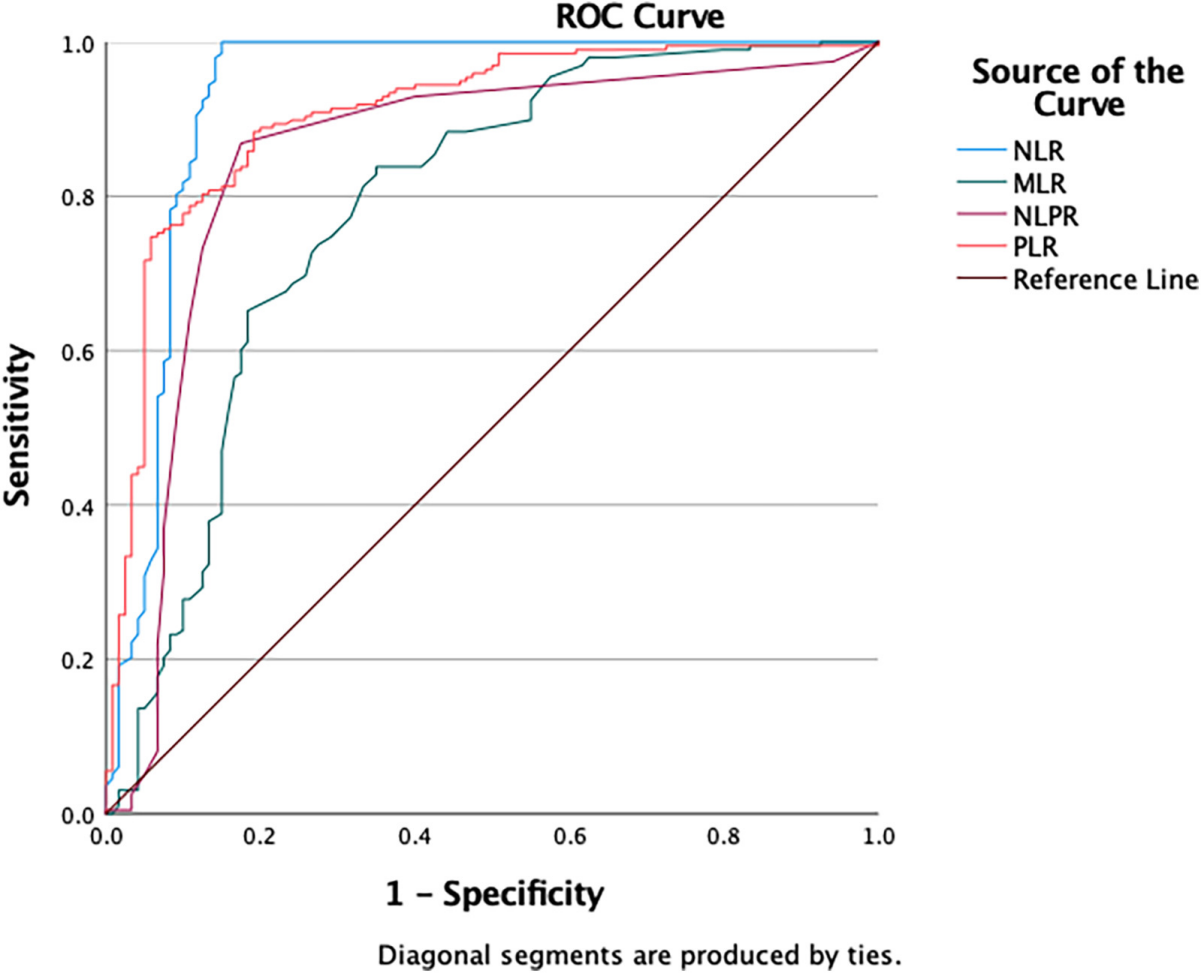
The ROC curves of classification efficacies of immune ratios.

The ROC curve analysis showed varied diagnostic abilities of immune ratios for sepsis detection. NLR (AUC = 0.932) and PLR (AUC = 0.907) had high predictive accuracy. MLR (AUC = 0.784) had limited discriminative power due to monocyte heterogeneity in chronic inflammation. NLPR (AUC = 0.848) showed better robustness by combining multiple parameters. These findings suggest NLR, PLR and NLPR could be effective biomarkers for sepsis diagnosis, while MLR needs additional inflammatory indicators for interpretation (Figure 2 and Table 2).

**Table 2:** Detection efficacy of immune ratios and laboratory tests for diagnosing sepsis.

Test result variable(s)	Area	*P*	95% Confidence interval		Cut off	Sensitivity	Specificity	Youden
			Lower bound	Upper bound				
NLR	0.932	0.000	0.894	0.969	1.17	1.000	0.850	0.85
MLR	0.784	0.000	0.728	0.840	0.31	0.838	0.650	0.49
NLPR	0.848	0.000	0.797	0.899	0.03	0.869	0.825	0.69
PLR	0.907	0.000	0.871	0.942	78.10	0.884	0.808	0.69
WBC	0.902	0.000	0.862	0.943	11.33	0.935	0.867	0.80
Neutrophil	0.919	0.000	0.881	0.957	4.87	0.935	0.875	0.81
Lymphocyte	0.755	0.000	0.701	0.809	2.79	0.792	0.632	0.42
Monocyte	0.623	0.000	0.559	0.687	1.19	0.527	0.750	0.28
Hemoglobin	0.666	0.000	0.604	0.728	133.50	0.392	0.900	0.29
PLT	0.879	0.000	0.841	0.918	295.50	0.596	0.983	0.58
CRP	0.923	0.000	0.882	0.963	9.71	0.974	0.817	0.79
PCT	0.772	0.000	0.712	0.832	0.06	0.968	0.550	0.52
ESR	0.875	0.000	0.829	0.921	9.92	0.897	0.800	0.70

### Efficacy evaluation of laboratory tests

**Figure 3:**
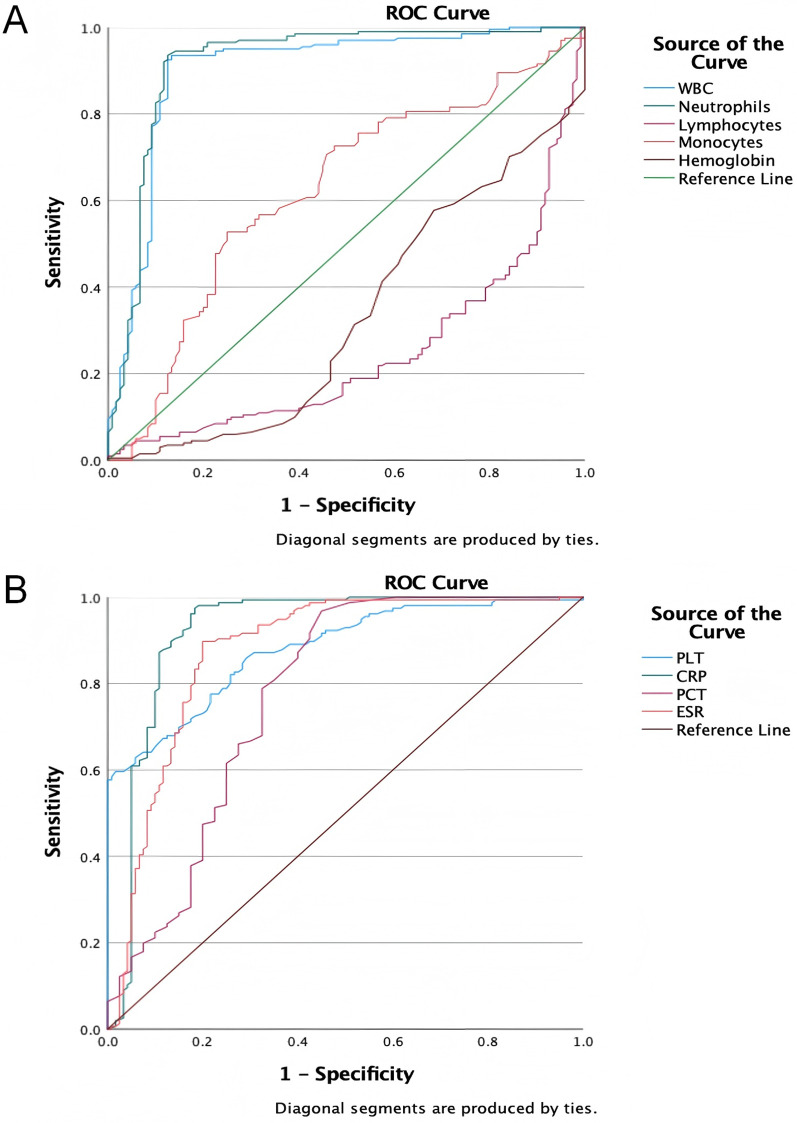
The ROC curves of laboratory tests for diagnosing sepsis. **(A)** The ROC curves of WBC, neutrophils, lymphocytes, monocytes and hemoglobin for diagnosing sepsis; **(B)** the ROC curves of PLT, CRP, PCT and ESR for diagnosing sepsis.

The ROC curve analysis of laboratory tests for sepsis diagnosis showed varied detection efficacy. WBC (AUC = 0.902) and Neutrophil (AUC = 0.919) demonstrated high diagnostic accuracy, with sensitivities of 0.935 and 0.935, and specificities of 0.867 and 0.875, respectively. CRP (AUC = 0.923) also showed strong predictive ability, with a sensitivity of 0.974 and a specificity of 0.817. In contrast, Lymphocyte (AUC = 0.755) and Monocyte (AUC = 0.623) had relatively lower discriminative power. PLT (AUC = 0.879) and ESR (AUC = 0.875) exhibited good diagnostic performance, with specificities of 0.983 and 0.800, respectively. PCT (AUC = 0.772) had moderate diagnostic ability. These findings suggest that WBC, Neutrophil, and CRP could be valuable biomarkers for sepsis diagnosis, while the utility of Lymphocyte, Monocyte, and Hemoglobin may be limited (Figures 3A,B and Table 2).

## Discussion

Immune cells are pivotal in the pathogenesis and progression of sepsis, with changes in their quantity and function significantly affecting the inflammatory and anti-inflammatory balance in the early stage of disease (24–26). The study systematically assessed the diagnostic value of different immune cell ratios and laboratory indicators in children with sepsis, revealing significant differences between sepsis patients and healthy controls in multiple immune parameters and laboratory test indicators.

The significant differences observed in various laboratory indicators, including WBC, neutrophil count, CRP, and PLT, highlight the potential of these parameters as biomarkers for sepsis. Notably, the decrease in lymphocyte count and monocyte count, along with the increase in the NLR, provide insights into the immune suppression state in sepsis. ROC curve analysis demonstrated that laboratory indicators such as neutrophils, CRP, and WBC, as well as immune cell ratio parameters like NLR and PLR, exhibited excellent diagnostic performance. These findings underscore the importance of multi-parameter monitoring in the early diagnosis of pediatric sepsis.

At the immune mechanism level, the imbalance in immune cells, characterized by lymphopenia and neutrophilia, is of particular concern. This imbalance may be related to the accelerated apoptosis of lymphocytes or their migration to lymphoid tissue due to cytokine storms (27). For instance, aerobic glycolysis mediated by pyruvate kinase M2 (PKM2) has been shown to promote inflammasome activation in macrophages via EIF2AK2 phosphorylation, exacerbating the release of pro-inflammatory cytokines like IL-1β and HMGB1 in sepsis (28). Although this study did not directly detect specific immune cell subsets, the significant increase in NLR indirectly suggests the pathological process of lymphocyte exhaustion. Additionally, the synergistic changes of increased platelet count, and decreased lymphocyte count may reflect the dual role of platelets in amplifying inflammation and immune regulation.

The innovation of this study lies in the systematic validation of the diagnostic value of easily obtainable parameters such as NLR and PLR in pediatric sepsis (AUC > 0.9), and the proposal of a new composite indicator, the NLPR (AUC = 0.848). Although CRP alone showed the highest diagnostic efficacy (AUC = 0.923), immune cell ratio parameters provide an additional dimension for interpreting the immune status. However, a meta-analysis of 28 studies revealed that CRP's pooled sensitivity (0.71) was lower than procalcitonin (PCT, 0.85) and combined PCT + CRP (0.91), underscoring its limited utility as a single marker (29). Our finding that NLR + CRP combination enhances diagnostic accuracy aligns with this, suggesting synergistic value of cellular and humoral inflammatory indicators.NLPR, by integrating information on neutrophil activation and monocyte function, may be more suitable for distinguishing immune-suppressed subtypes (20).

It should be noted that while no significant differences in specific lymphocyte subsets were observed between the SP and HC groups, parameters such as platelets (PLT) and hemoglobin showed statistical differences, which may be related to inflammatory anemia and bone marrow stress responses during the course of sepsis (30). These findings suggest that the interpretation of single indicators needs to be combined with clinical context.

The limitations of this study include the single-center retrospective design, which may limit the generalizability of the conclusions. Additionally, the absence of cell function tests makes it difficult to clarify the mechanistic associations, and the small sample size may affect the reliability of subgroup analyses (20). Future research should conduct multi-center prospective studies, combined with technologies such as single-cell sequencing, to further validate the clinical value of immune cell ratio parameters (31).

## Conclusion

In conclusion, our study reveals that combining immune cell ratios and CRP significantly enhances early detection of pediatric sepsis, improving clinical outcomes.

## Data Availability

The original contributions presented in the study are included in the article/Supplementary Material, further inquiries can be directed to the corresponding author.
